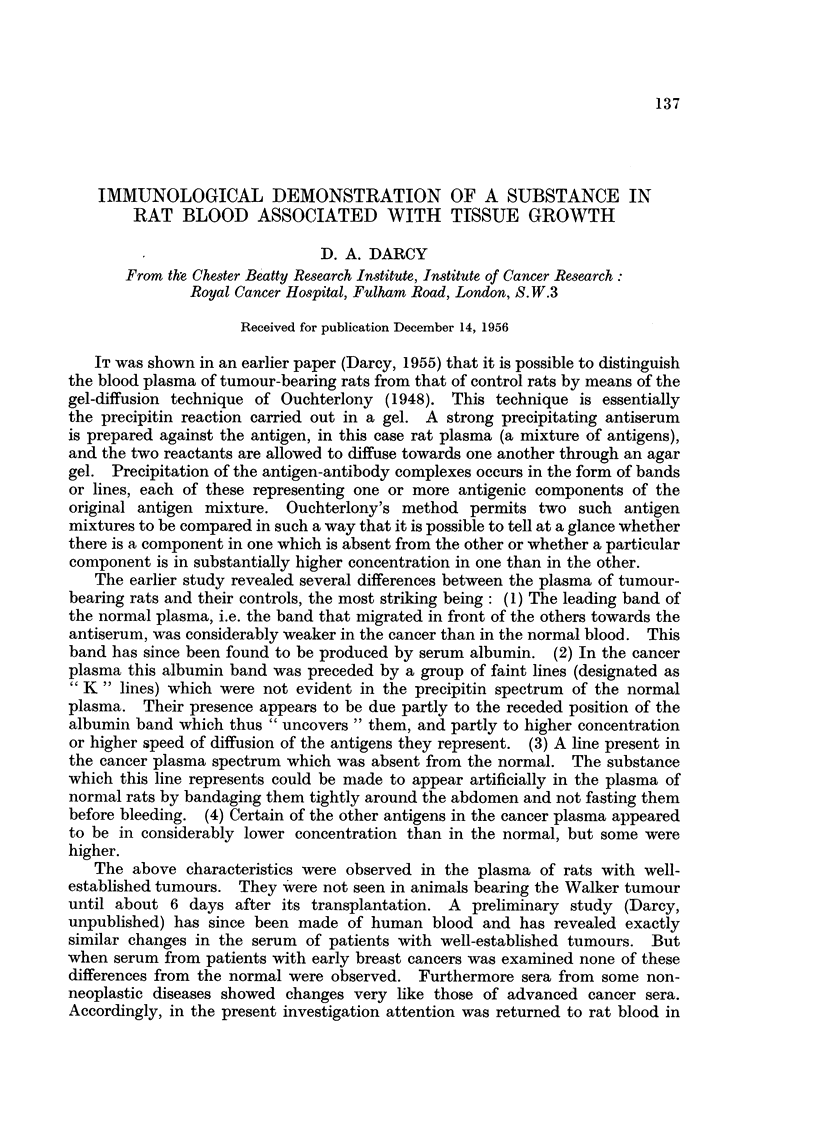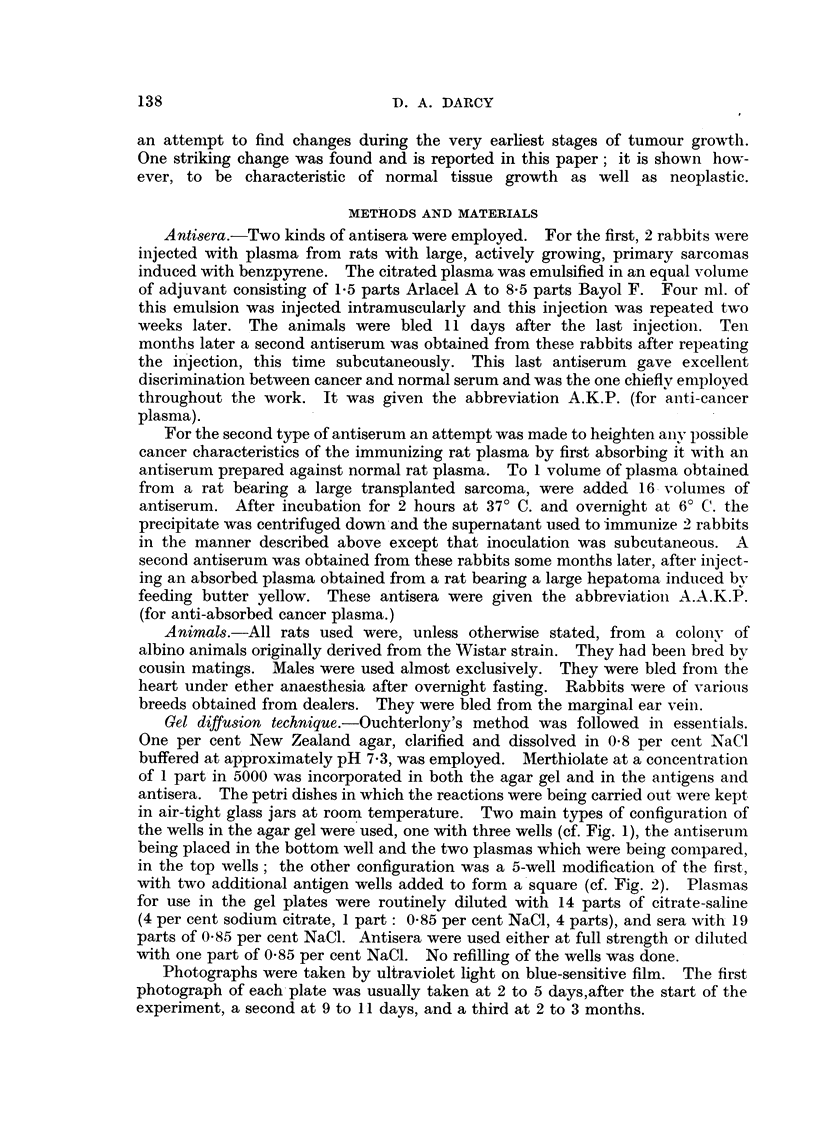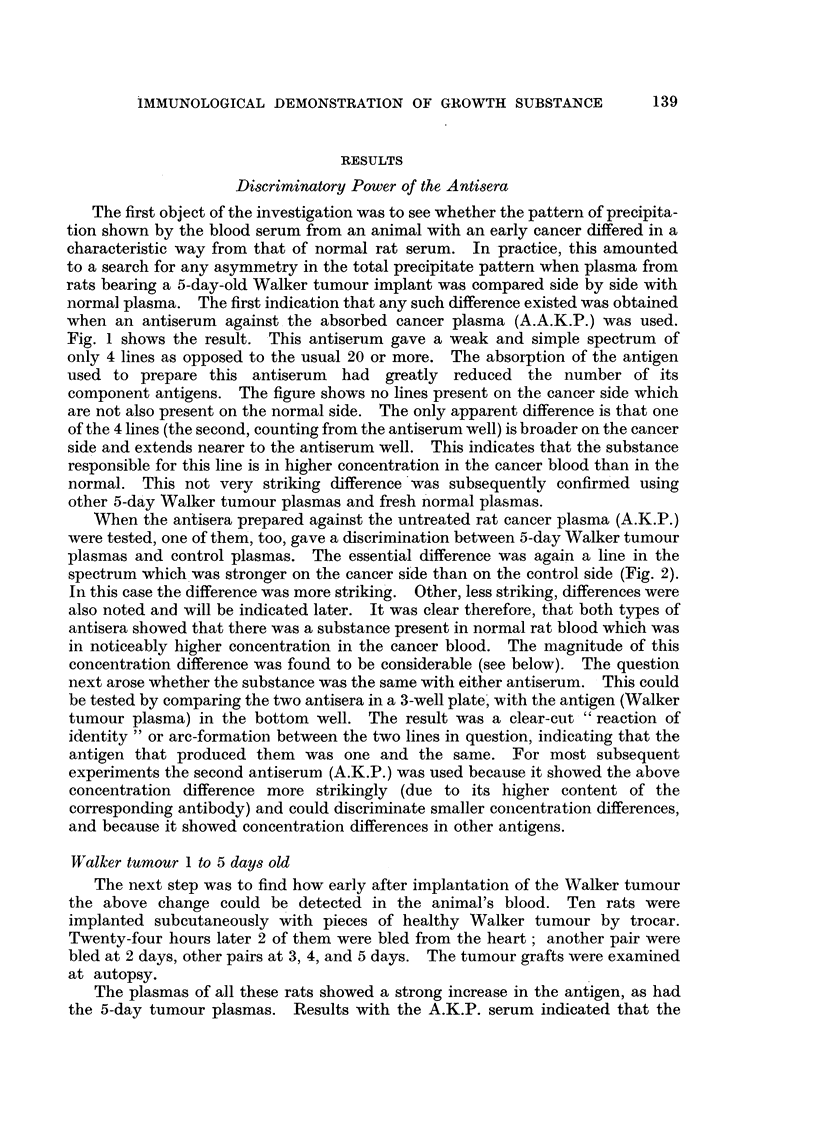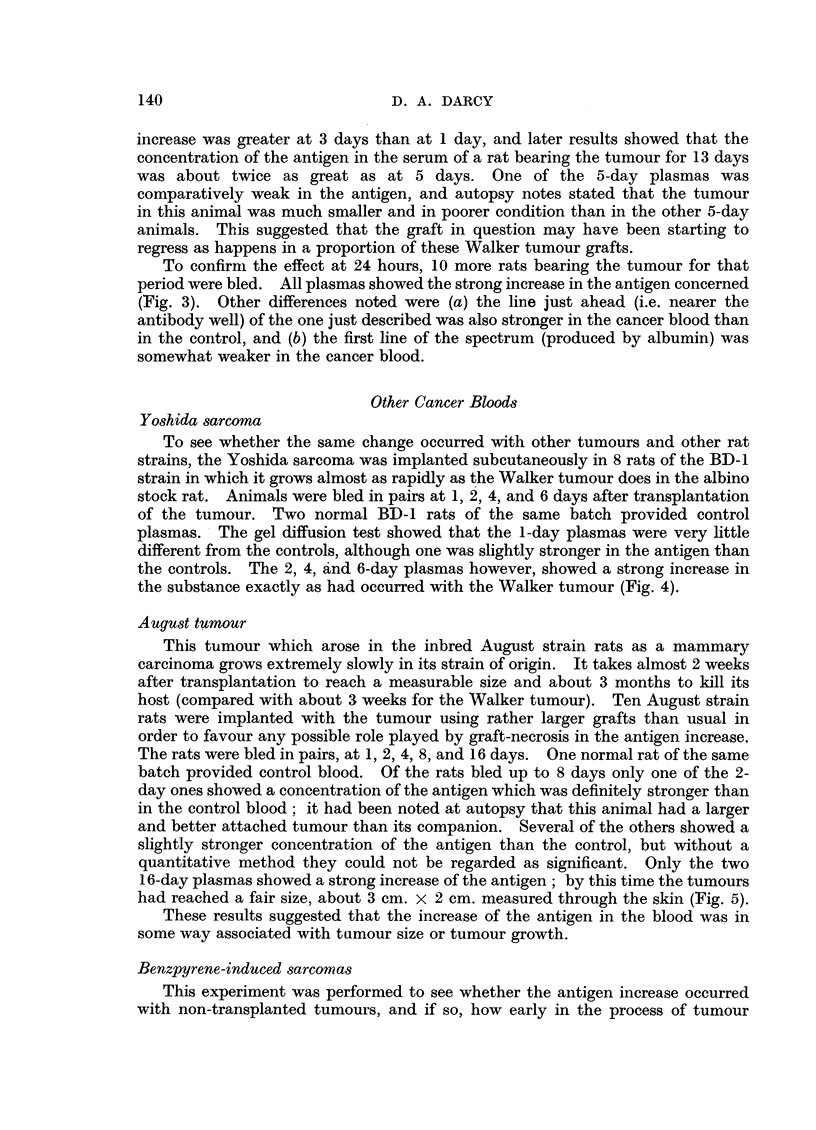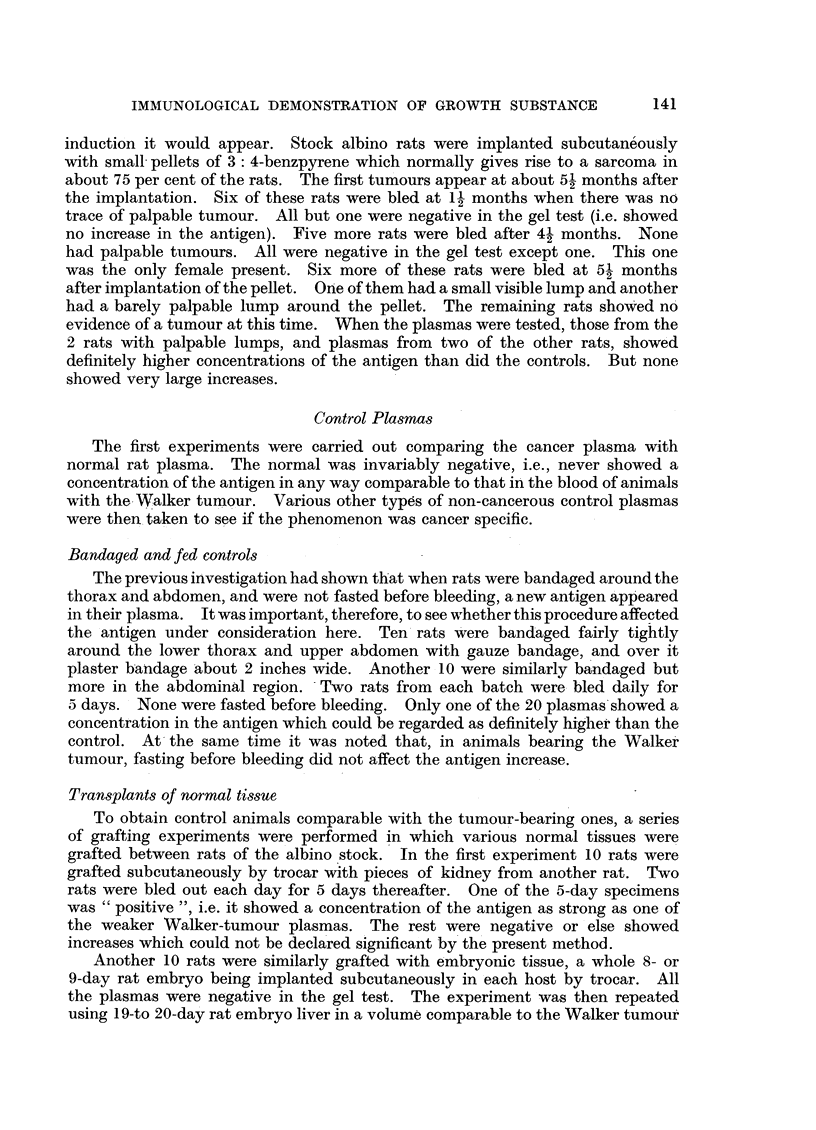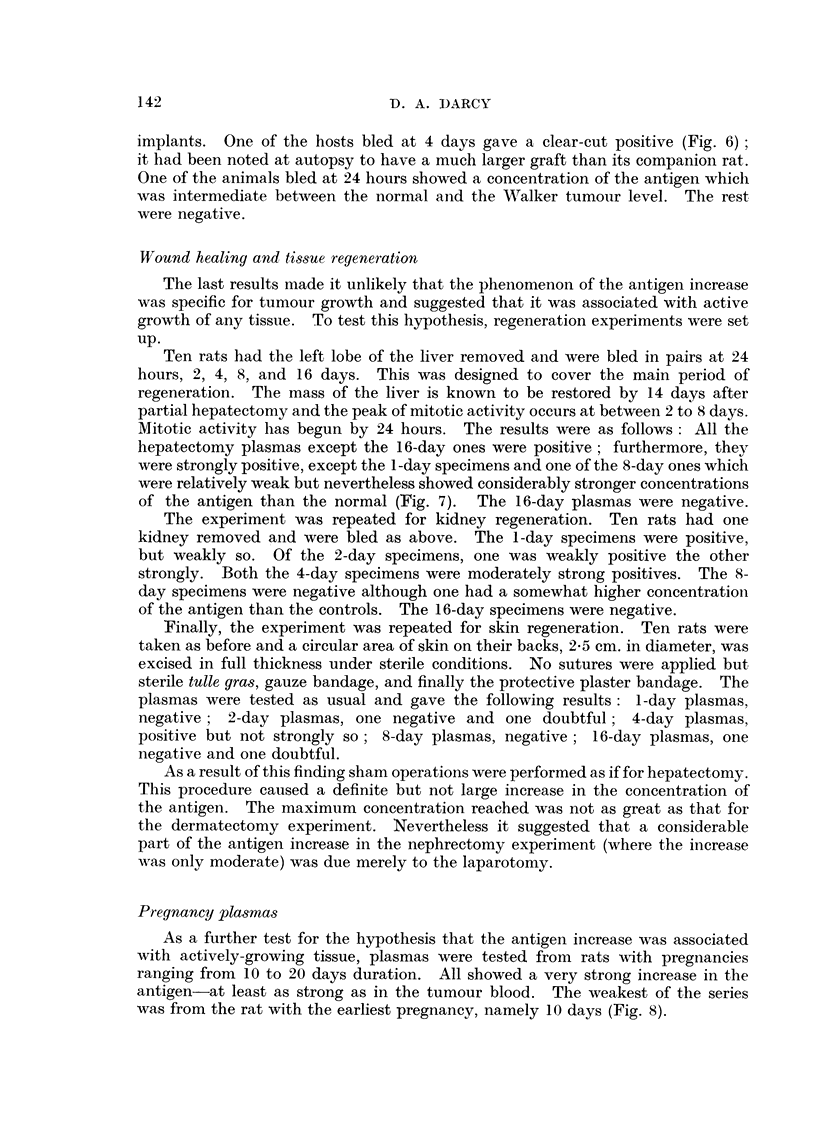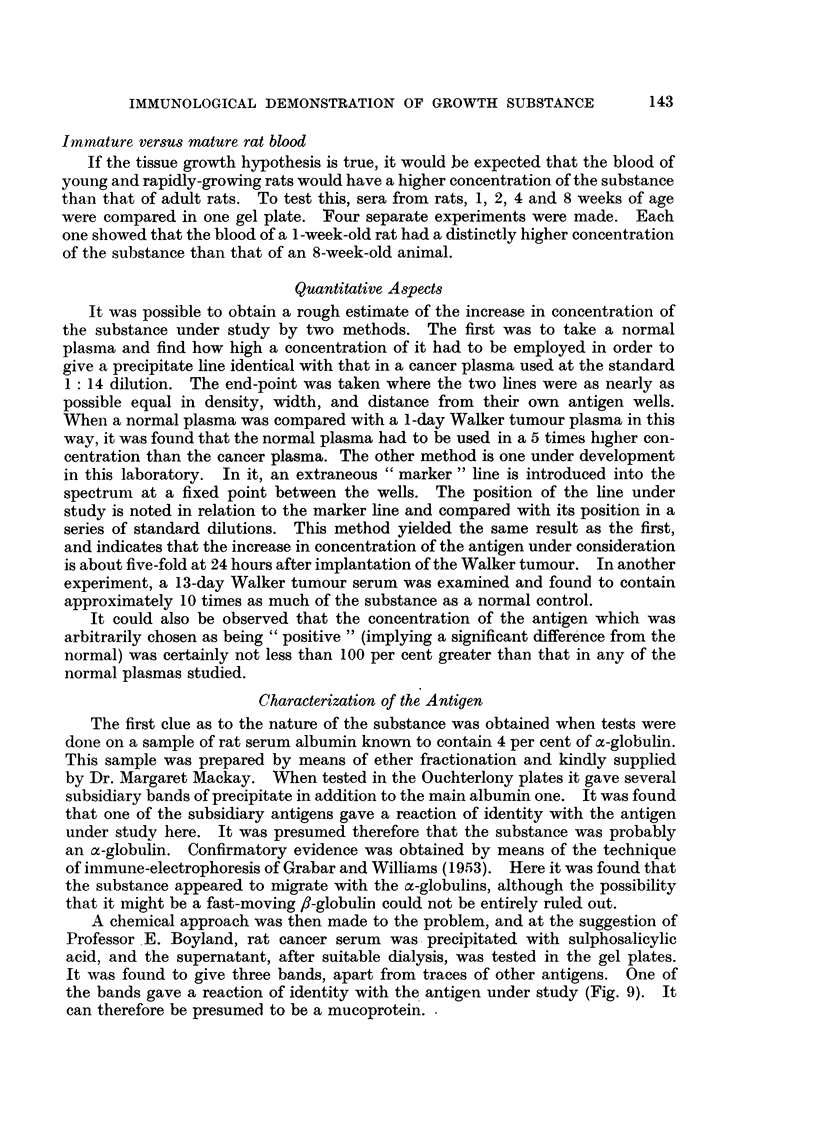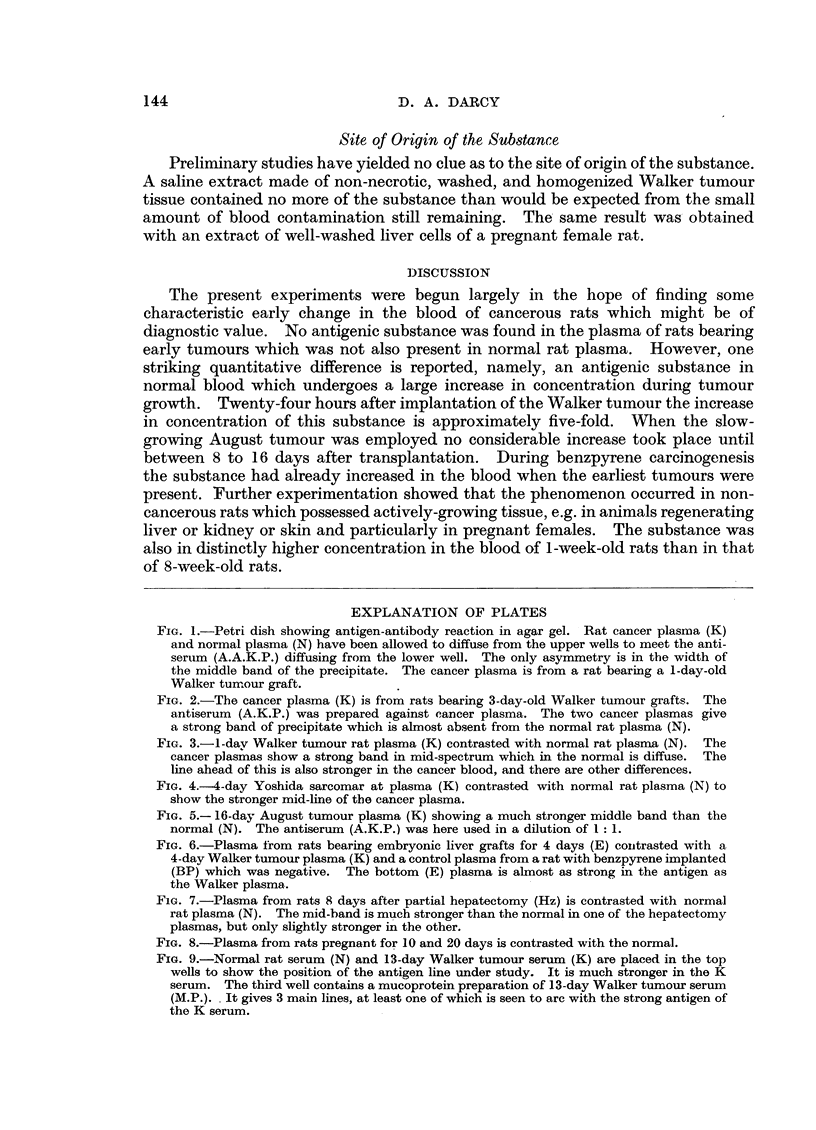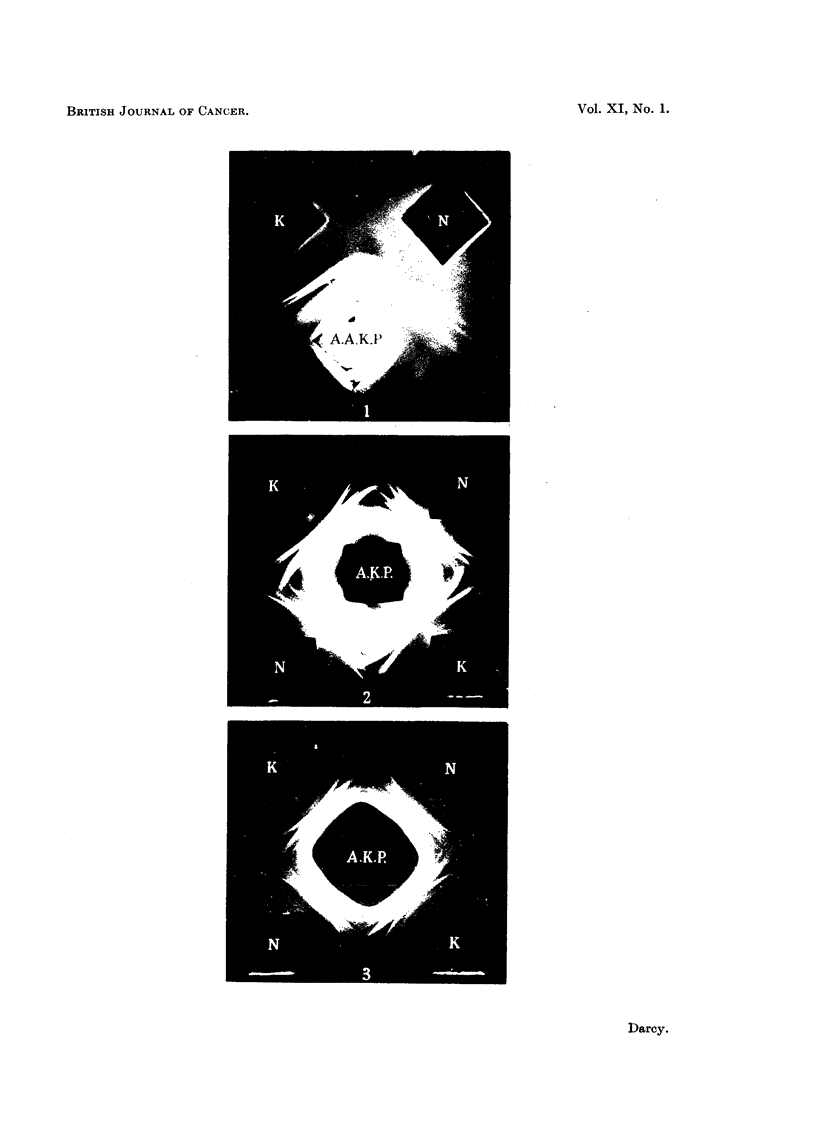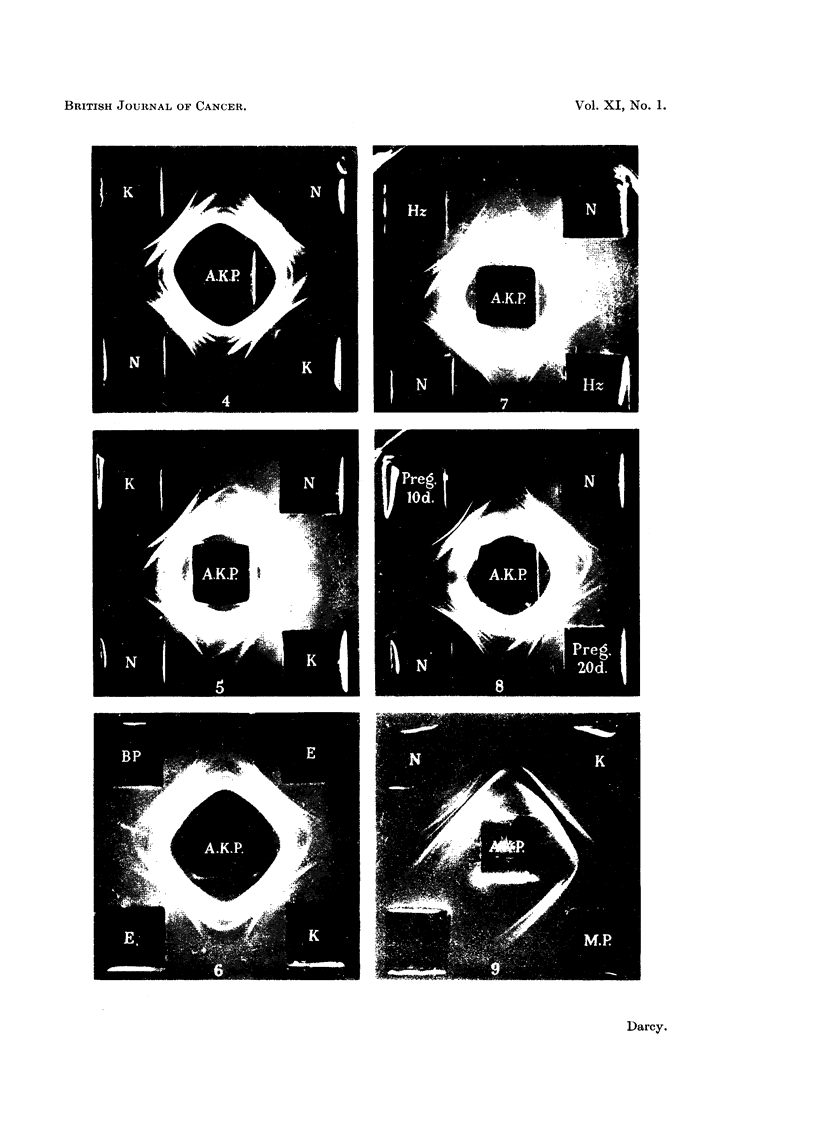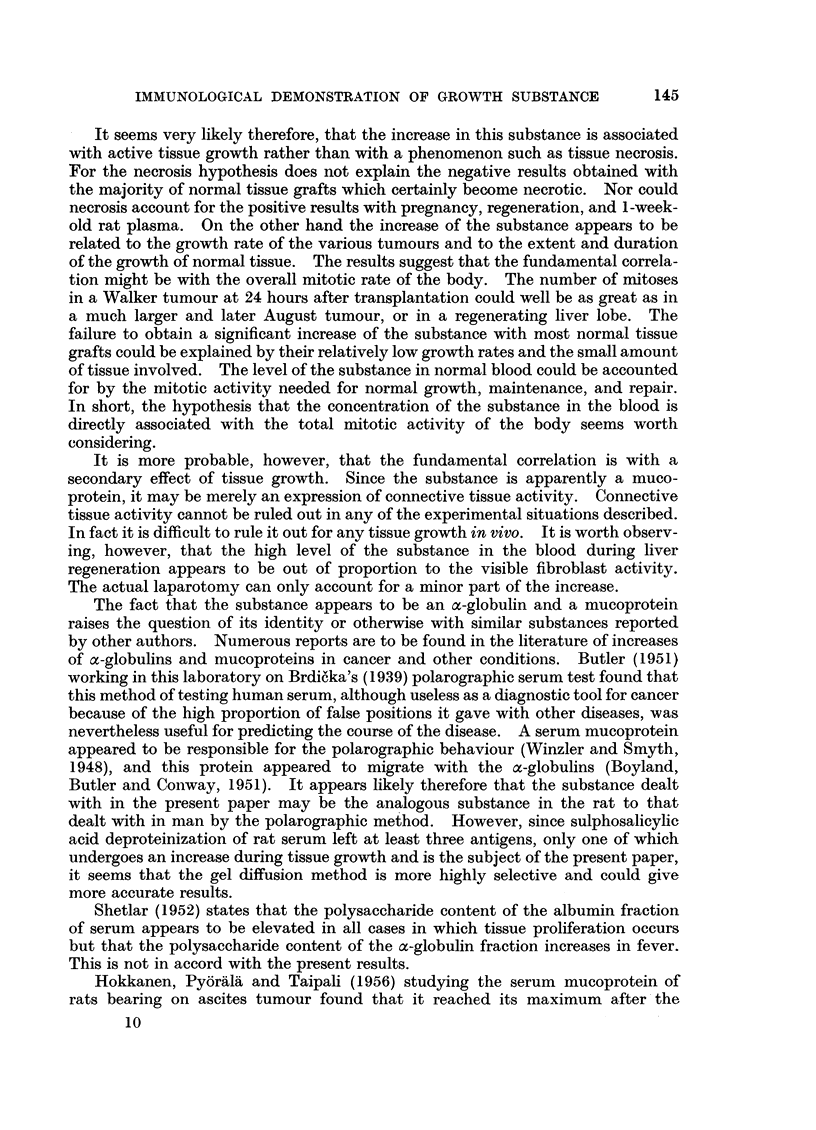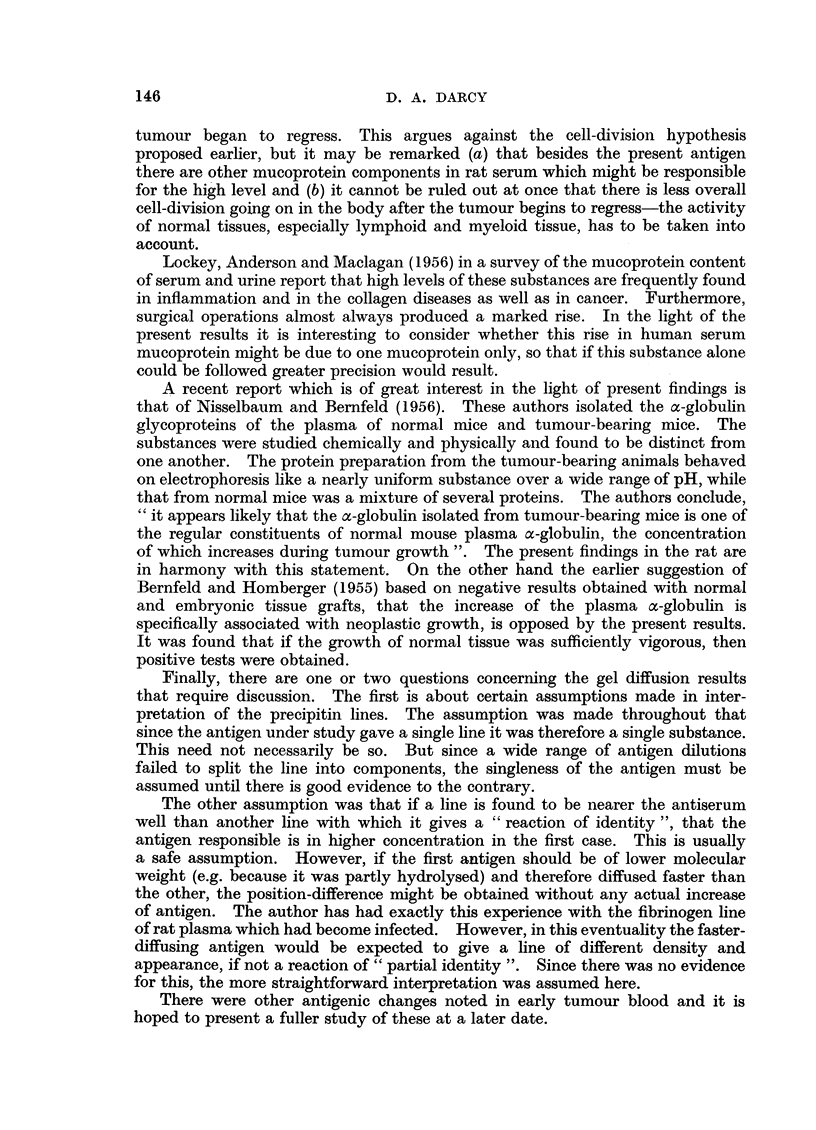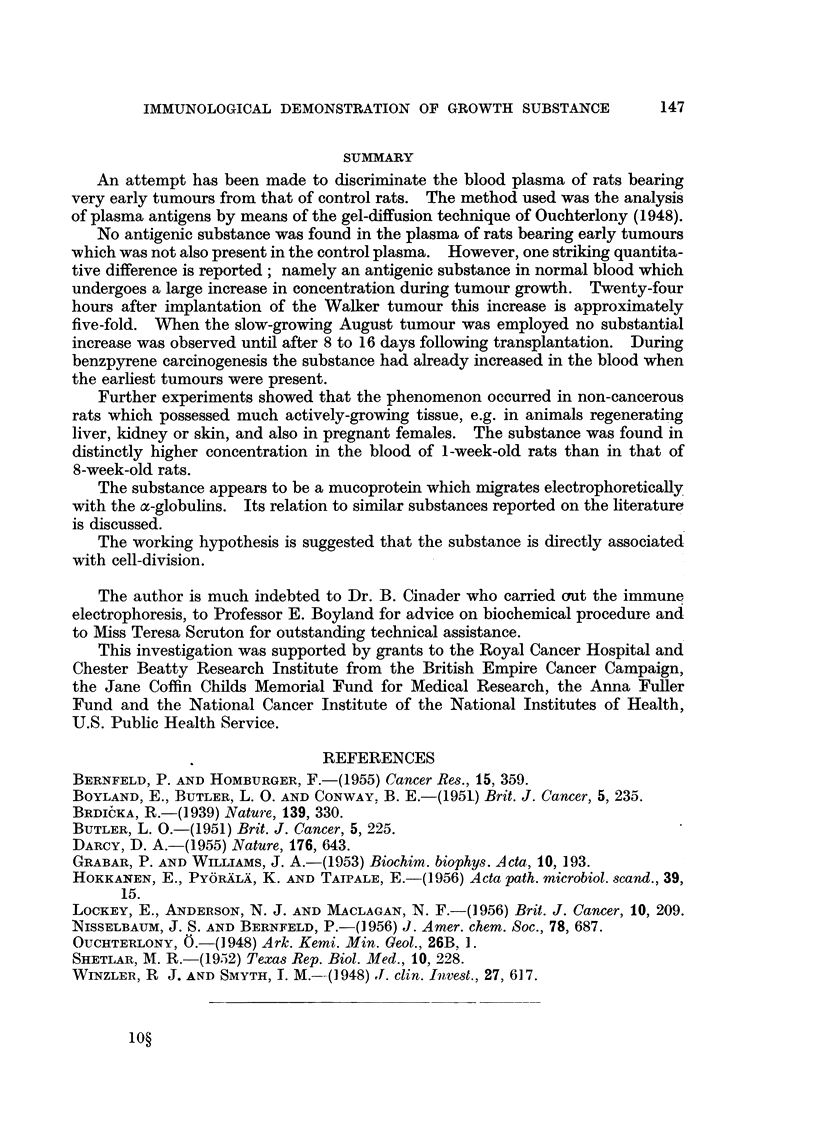# Immunological Demonstration of a Substance in Rat Blood Associated with Tissue Growth

**DOI:** 10.1038/bjc.1957.20

**Published:** 1957-03

**Authors:** D. A. Darcy

## Abstract

**Images:**


### IMMUNOLOGICAL DEMONSTRATION OF A SUBSTANCE IN RAT BLOOD ASSOCIATED WITH TISSUE GROWTH

D. A. DARCY

*From the Chester Beatty Research Institute, Institute of Cancer Research : Royal Cancer Hospital, Fulham Road, London, S.W.3*

Received for publication December 14, 1956

IT was shown in an earlier paper (Darcy, 1955) that it is possible to distinguish the blood plasma of tumour-bearing rats from that of control rats by means of the gel-diffusion technique of Ouchterlony (1948). This technique is essentially the precipitin reaction carried out in a gel. A strong precipitating antiserum is prepared against the antigen, in this case rat plasma (a mixture of antigens), and the two reactants are allowed to diffuse towards one another through an agar gel. Precipitation of the antigen-antibody complexes occurs in the form of bands or lines, each of these representing one or more antigenic components of the original antigen mixture. Ouchterlony's method permits two such antigen mixtures to be compared in such a way that it is possible to tell at a glance whether there is a component in one which is absent from the other or whether a particular component is in substantially higher concentration in one than in the other.

The earlier study revealed several differences between the plasma of tumour-bearing rats and their controls, the most striking being : (1) The leading band of the normal plasma, i.e. the band that migrated in front of the others towards the antiserum, was considerably weaker in the cancer than in the normal blood. This band has since been found to be produced by serum albumin. (2) In the cancer plasma this albumin band was preceded by a group of faint lines (designated as " K " lines) which were not evident in the precipitin spectrum of the normal plasma. Their presence appears to be due partly to the receded position of the albumin band which thus " uncovers " them, and partly to higher concentration or higher speed of diffusion of the antigens they represent. (3) A line present in the cancer plasma spectrum which was absent from the normal. The substance which this line represents could be made to appear artificially in the plasma of normal rats by bandaging them tightly around the abdomen and not fasting them before bleeding. (4) Certain of the other antigens in the cancer plasma appeared to be in considerably lower concentration than in the normal, but some were higher.

The above characteristics were observed in the plasma of rats with well-established tumours. They were not seen in animals bearing the Walker tumour until about 6 days after its transplantation. A preliminary study (Darcy, unpublished) has since been made of human blood and has revealed exactly similar changes in the serum of patients with well-established tumours. But when serum from patients with early breast cancers was examined none of these differences from the normal were observed. Furthermore sera from some non-neoplastic diseases showed changes very like those of advanced cancer sera. Accordingly, in the present investigation attention was returned to rat blood in an attempt to find changes during the very earliest stages of tumour growth. One striking change was found and is reported in this paper ; it is shown however, to be characteristic of normal tissue growth as well as neoplastic.

### METHODS AND MATERIALS

*Antisera.*—Two kinds of antisera were employed. For the first, 2 rabbits were injected with plasma from rats with large, actively growing, primary sarcomas induced with benzpyrene. The citrated plasma was emulsified in an equal volume of adjuvant consisting of 1·5 parts Arlacel A to 8·5 parts Bayol F. Four ml. of this emulsion was injected intramuscularly and this injection was repeated two weeks later. The animals were bled 11 days after the last injection. Ten months later a second antiserum was obtained from these rabbits after repeating the injection, this time subcutaneously. This last antiserum gave excellent discrimination between cancer and normal serum and was the one chiefly employed throughout the work. It was given the abbreviation A.K.P. (for anti-cancer plasma).

For the second type of antiserum an attempt was made to heighten any possible cancer characteristics of the immunizing rat plasma by first absorbing it with an antiserum prepared against normal rat plasma. To 1 volume of plasma obtained from a rat bearing a large transplanted sarcoma, were added 16 volumes of antiserum. After incubation for 2 hours at 37° C. and overnight at 6° C. the precipitate was centrifuged down and the supernatant used to immunize 2 rabbits in the manner described above except that inoculation was subcutaneous. A second antiserum was obtained from these rabbits some months later, after injecting an absorbed plasma obtained from a rat bearing a large hepatoma induced by feeding butter yellow. These antisera were given the abbreviation A.A.K.P. (for anti-absorbed cancer plasma.)

*Animals.*—All rats used were, unless otherwise stated, from a colony of albino animals originally derived from the Wistar strain. They had been bred by cousin matings. Males were used almost exclusively. They were bled from the heart under ether anaesthesia after overnight fasting. Rabbits were of various breeds obtained from dealers. They were bled from the marginal ear vein.

*Gel diffusion technique.*—Ouchterlony's method was followed in essentials. One per cent New Zealand agar, clarified and dissolved in 0·8 per cent NaCl buffered at approximately pH 7·3, was employed. Merthiolate at a concentration of 1 part in 5000 was incorporated in both the agar gel and in the antigens and antisera. The petri dishes in which the reactions were being carried out were kept in air-tight glass jars at room temperature. Two main types of configuration of the wells in the agar gel were used, one with three wells (cf. Fig. 1), the antiserum being placed in the bottom well and the two plasmas which were being compared, in the top wells ; the other configuration was a 5-well modification of the first, with two additional antigen wells added to form a square (cf. Fig. 2). Plasmas for use in the gel plates were routinely diluted with 14 parts of citrate-saline (4 per cent sodium citrate, 1 part : 0·85 per cent NaCl, 4 parts), and sera with 19 parts of 0·85 per cent NaCl. Antisera were used either at full strength or diluted with one part of 0·85 per cent NaCl. No refilling of the wells was done.

Photographs were taken by ultraviolet light on blue-sensitive film. The first photograph of each plate was usually taken at 2 to 5 days,after the start of the experiment, a second at 9 to 11 days, and a third at 2 to 3 months.

### RESULTS

### *Discriminatory Power of the Antisera*

The first object of the investigation was to see whether the pattern of precipitation shown by the blood serum from an animal with an early cancer differed in a characteristic way from that of normal rat serum. In practice, this amounted to a search for any asymmetry in the total precipitate pattern when plasma from rats bearing a 5-day-old Walker tumour implant was compared side by side with normal plasma. The first indication that any such difference existed was obtained when an antiserum against the absorbed cancer plasma (A.A.K.P.) was used. Fig. 1 shows the result. This antiserum gave a weak and simple spectrum of only 4 lines as opposed to the usual 20 or more. The absorption of the antigen used to prepare this antiserum had greatly reduced the number of its component antigens. The figure shows no lines present on the cancer side which are not also present on the normal side. The only apparent difference is that one of the 4 lines (the second, counting from the antiserum well) is broader on the cancer side and extends nearer to the antiserum well. This indicates that the substance responsible for this line is in higher concentration in the cancer blood than in the normal. This not very striking difference was subsequently confirmed using other 5-day Walker tumour plasmas and fresh normal plasmas.

When the antisera prepared against the untreated rat cancer plasma (A.K.P.) were tested, one of them, too, gave a discrimination between 5-day Walker tumour plasmas and control plasmas. The essential difference was again a line in the spectrum which was stronger on the cancer side than on the control side (Fig. 2). In this case the difference was more striking. Other, less striking, differences were also noted and will be indicated later. It was clear therefore, that both types of antisera showed that there was a substance present in normal rat blood which was in noticeably higher concentration in the cancer blood. The magnitude of this concentration difference was found to be considerable (see below). The question next arose whether the substance was the same with either antiserum. This could be tested by comparing the two antisera in a 3-well plate, with the antigen (Walker tumour plasma) in the bottom well. The result was a clear-cut " reaction of identity " or arc-formation between the two lines in question, indicating that the antigen that produced them was one and the same. For most subsequent experiments the second antiserum (A.K.P.) was used because it showed the above concentration difference more strikingly (due to its higher content of the corresponding antibody) and could discriminate smaller concentration differences, and because it showed concentration differences in other antigens.

### *Walker tumour* 1 *to* 5 *days old*

The next step was to find how early after implantation of the Walker tumour the above change could be detected in the animal's blood. Ten rats were implanted subcutaneously with pieces of healthy Walker tumour by trocar. Twenty-four hours later 2 of them were bled from the heart ; another pair were bled at 2 days, other pairs at 3, 4, and 5 days. The tumour grafts were examined at autopsy.

The plasmas of all these rats showed a strong increase in the antigen, as had the 5-day tumour plasmas. Results with the A.K.P. serum indicated that the increase was greater at 3 days than at 1 day, and later results showed that the concentration of the antigen in the serum of a rat bearing the tumour for 13 days was about twice as great as at 5 days. One of the 5-day plasmas was comparatively weak in the antigen, and autopsy notes stated that the tumour in this animal was much smaller and in poorer condition than in the other 5-day animals. This suggested that the graft in question may have been starting to regress as happens in a proportion of these Walker tumour grafts.

To confirm the effect at 24 hours, 10 more rats bearing the tumour for that period were bled. All plasmas showed the strong increase in the antigen concerned (Fig. 3). Other differences noted were (*a*) the line just ahead (i.e. nearer the antibody well) of the one just described was also stronger in the cancer blood than in the control, and (*b*) the first line of the spectrum (produced by albumin) was somewhat weaker in the cancer blood.

### *Other Cancer Bloods*

### *Yoshida sarcoma*

To see whether the same change occurred with other tumours and other rat strains, the Yoshida sarcoma was implanted subcutaneously in 8 rats of the BD-1 strain in which it grows almost as rapidly as the Walker tumour does in the albino stock rat. Animals were bled in pairs at 1, 2, 4, and 6 days after transplantation of the tumour. Two normal BD-1 rats of the same batch provided control plasmas. The gel diffusion test showed that the 1-day plasmas were very little different from the controls, although one was slightly stronger in the antigen than the controls. The 2, 4, and 6-day plasmas however, showed a strong increase in the substance exactly as had occurred with the Walker tumour (Fig. 4).

### *August tumour*

This tumour which arose in the inbred August strain rats as a mammary carcinoma grows extremely slowly in its strain of origin. It takes almost 2 weeks after transplantation to reach a measurable size and about 3 months to kill its host (compared with about 3 weeks for the Walker tumour). Ten August strain rats were implanted with the tumour using rather larger grafts than usual in order to favour any possible role played by graft-necrosis in the antigen increase. The rats were bled in pairs, at 1, 2, 4, 8, and 16 days. One normal rat of the same batch provided control blood. Of the rats bled up to 8 days only one of the 2-day ones showed a concentration of the antigen which was definitely stronger than in the control blood ; it had been noted at autopsy that this animal had a larger and better attached tumour than its companion. Several of the others showed a slightly stronger concentration of the antigen than the control, but without a quantitative method they could not be regarded as significant. Only the two 16-day plasmas showed a strong increase of the antigen ; by this time the tumours had reached a fair size, about 3 cm. $\times$ 2 cm. measured through the skin (Fig. 5).

These results suggested that the increase of the antigen in the blood was in some way associated with tumour size or tumour growth.

### *Benzpyrene-induced sarcomas*

This experiment was performed to see whether the antigen increase occurred with non-transplanted tumours, and if so, how early in the process of tumour induction it would appear. Stock albino rats were implanted subcutaneously with small pellets of 3 : 4-benzpyrene which normally gives rise to a sarcoma in about 75 per cent of the rats. The first tumours appear at about $5\frac{1}{2}$ months after the implantation. Six of these rats were bled at $1\frac{1}{2}$ months when there was no trace of palpable tumour. All but one were negative in the gel test (i.e. showed no increase in the antigen). Five more rats were bled after $4\frac{1}{2}$ months. None had palpable tumours. All were negative in the gel test except one. This one was the only female present. Six more of these rats were bled at $5\frac{1}{2}$ months after implantation of the pellet. One of them had a small visible lump and another had a barely palpable lump around the pellet. The remaining rats showed no evidence of a tumour at this time. When the plasmas were tested, those from the 2 rats with palpable lumps, and plasmas from two of the other rats, showed definitely higher concentrations of the antigen than did the controls. But none showed very large increases.

### *Control Plasmas*

The first experiments were carried out comparing the cancer plasma with normal rat plasma. The normal was invariably negative, i.e., never showed a concentration of the antigen in any way comparable to that in the blood of animals with the Walker tumour. Various other types of non-cancerous control plasmas were then taken to see if the phenomenon was cancer specific.

*Bandaged and fed controls*

The previous investigation had shown that when rats were bandaged around the thorax and abdomen, and were not fasted before bleeding, a new antigen appeared in their plasma. It was important, therefore, to see whether this procedure affected the antigen under consideration here. Ten rats were bandaged fairly tightly around the lower thorax and upper abdomen with gauze bandage, and over it plaster bandage about 2 inches wide. Another 10 were similarly bandaged but more in the abdominal region. Two rats from each batch were bled daily for 5 days. None were fasted before bleeding. Only one of the 20 plasmas showed a concentration in the antigen which could be regarded as definitely higher than the control. At the same time it was noted that, in animals bearing the Walker tumour, fasting before bleeding did not affect the antigen increase.

*Transplants of normal tissue*

To obtain control animals comparable with the tumour-bearing ones, a series of grafting experiments were performed in which various normal tissues were grafted between rats of the albino stock. In the first experiment 10 rats were grafted subcutaneously by trocar with pieces of kidney from another rat. Two rats were bled out each day for 5 days thereafter. One of the 5-day specimens was " positive ", i.e. it showed a concentration of the antigen as strong as one of the weaker Walker-tumour plasmas. The rest were negative or else showed increases which could not be declared significant by the present method.

Another 10 rats were similarly grafted with embryonic tissue, a whole 8- or 9-day rat embryo being implanted subcutaneously in each host by trocar. All the plasmas were negative in the gel test. The experiment was then repeated using 19-to 20-day rat embryo liver in a volume comparable to the Walker tumour implants. One of the hosts bled at 4 days gave a clear-cut positive (Fig. 6); it had been noted at autopsy to have a much larger graft than its companion rat. One of the animals bled at 24 hours showed a concentration of the antigen which was intermediate between the normal and the Walker tumour level. The rest were negative.

*Wound healing and tissue regeneration*

The last results made it unlikely that the phenomenon of the antigen increase was specific for tumour growth and suggested that it was associated with active growth of any tissue. To test this hypothesis, regeneration experiments were set up.

Ten rats had the left lobe of the liver removed and were bled in pairs at 24 hours, 2, 4, 8, and 16 days. This was designed to cover the main period of regeneration. The mass of the liver is known to be restored by 14 days after partial hepatectomy and the peak of mitotic activity occurs at between 2 to 8 days. Mitotic activity has begun by 24 hours. The results were as follows: All the hepatectomy plasmas except the 16-day ones were positive; furthermore, they were strongly positive, except the 1-day specimens and one of the 8-day ones which were relatively weak but nevertheless showed considerably stronger concentrations of the antigen than the normal (Fig. 7). The 16-day plasmas were negative.

The experiment was repeated for kidney regeneration. Ten rats had one kidney removed and were bled as above. The 1-day specimens were positive, but weakly so. Of the 2-day specimens, one was weakly positive the other strongly. Both the 4-day specimens were moderately strong positives. The 8-day specimens were negative although one had a somewhat higher concentration of the antigen than the controls. The 16-day specimens were negative.

Finally, the experiment was repeated for skin regeneration. Ten rats were taken as before and a circular area of skin on their backs, 2·5 cm. in diameter, was excised in full thickness under sterile conditions. No sutures were applied but sterile *tulle gras*, gauze bandage, and finally the protective plaster bandage. The plasmas were tested as usual and gave the following results: 1-day plasmas, negative; 2-day plasmas, one negative and one doubtful; 4-day plasmas, positive but not strongly so; 8-day plasmas, negative; 16-day plasmas, one negative and one doubtful.

As a result of this finding sham operations were performed as if for hepatectomy. This procedure caused a definite but not large increase in the concentration of the antigen. The maximum concentration reached was not as great as that for the dermatectomy experiment. Nevertheless it suggested that a considerable part of the antigen increase in the nephrectomy experiment (where the increase was only moderate) was due merely to the laparotomy.

*Pregnancy plasmas*

As a further test for the hypothesis that the antigen increase was associated with actively-growing tissue, plasmas were tested from rats with pregnancies ranging from 10 to 20 days duration. All showed a very strong increase in the antigen—at least as strong as in the tumour blood. The weakest of the series was from the rat with the earliest pregnancy, namely 10 days (Fig. 8).

*Immature versus mature rat blood*

If the tissue growth hypothesis is true, it would be expected that the blood of young and rapidly-growing rats would have a higher concentration of the substance than that of adult rats. To test this, sera from rats, 1, 2, 4 and 8 weeks of age were compared in one gel plate. Four separate experiments were made. Each one showed that the blood of a 1-week-old rat had a distinctly higher concentration of the substance than that of an 8-week-old animal.

### *Quantitative Aspects*

It was possible to obtain a rough estimate of the increase in concentration of the substance under study by two methods. The first was to take a normal plasma and find how high a concentration of it had to be employed in order to give a precipitate line identical with that in a cancer plasma used at the standard 1 : 14 dilution. The end-point was taken where the two lines were as nearly as possible equal in density, width, and distance from their own antigen wells. When a normal plasma was compared with a 1-day Walker tumour plasma in this way, it was found that the normal plasma had to be used in a 5 times higher concentration than the cancer plasma. The other method is one under development in this laboratory. In it, an extraneous " marker " line is introduced into the spectrum at a fixed point between the wells. The position of the line under study is noted in relation to the marker line and compared with its position in a series of standard dilutions. This method yielded the same result as the first, and indicates that the increase in concentration of the antigen under consideration is about five-fold at 24 hours after implantation of the Walker tumour. In another experiment, a 13-day Walker tumour serum was examined and found to contain approximately 10 times as much of the substance as a normal control.

It could also be observed that the concentration of the antigen which was arbitrarily chosen as being " positive " (implying a significant difference from the normal) was certainly not less than 100 per cent greater than that in any of the normal plasmas studied.

### *Characterization of the Antigen*

The first clue as to the nature of the substance was obtained when tests were done on a sample of rat serum albumin known to contain 4 per cent of $\alpha$-globulin. This sample was prepared by means of ether fractionation and kindly supplied by Dr. Margaret Mackay. When tested in the Ouchterlony plates it gave several subsidiary bands of precipitate in addition to the main albumin one. It was found that one of the subsidiary antigens gave a reaction of identity with the antigen under study here. It was presumed therefore that the substance was probably an $\alpha$-globulin. Confirmatory evidence was obtained by means of the technique of immune-electrophoresis of Grabar and Williams (1953). Here it was found that the substance appeared to migrate with the $\alpha$-globulins, although the possibility that it might be a fast-moving $\beta$-globulin could not be entirely ruled out.

A chemical approach was then made to the problem, and at the suggestion of Professor E. Boyland, rat cancer serum was precipitated with sulphosalicylic acid, and the supernatant, after suitable dialysis, was tested in the gel plates. It was found to give three bands, apart from traces of other antigens. One of the bands gave a reaction of identity with the antigen under study (Fig. 9). It can therefore be presumed to be a mucoprotein.

*Site of Origin of the Substance*

Preliminary studies have yielded no clue as to the site of origin of the substance. A saline extract made of non-necrotic, washed, and homogenized Walker tumour tissue contained no more of the substance than would be expected from the small amount of blood contamination still remaining. The same result was obtained with an extract of well-washed liver cells of a pregnant female rat.

### DISCUSSION

The present experiments were begun largely in the hope of finding some characteristic early change in the blood of cancerous rats which might be of diagnostic value. No antigenic substance was found in the plasma of rats bearing early tumours which was not also present in normal rat plasma. However, one striking quantitative difference is reported, namely, an antigenic substance in normal blood which undergoes a large increase in concentration during tumour growth. Twenty-four hours after implantation of the Walker tumour the increase in concentration of this substance is approximately five-fold. When the slow-growing August tumour was employed no considerable increase took place until between 8 to 16 days after transplantation. During benzpyrene carcinogenesis the substance had already increased in the blood when the earliest tumours were present. Further experimentation showed that the phenomenon occurred in non-cancerous rats which possessed actively-growing tissue, e.g. in animals regenerating liver or kidney or skin and particularly in pregnant females. The substance was also in distinctly higher concentration in the blood of 1-week-old rats than in that of 8-week-old rats.

---

### EXPLANATION OF PLATES

FIG. 1.—Petri dish showing antigen-antibody reaction in agar gel. Rat cancer plasma (K) and normal plasma (N) have been allowed to diffuse from the upper wells to meet the anti-serum (A.A.K.P.) diffusing from the lower well. The only asymmetry is in the width of the middle band of the precipitate. The cancer plasma is from a rat bearing a 1-day-old Walker tumour graft.

FIG. 2.—The cancer plasma (K) is from rats bearing 3-day-old Walker tumour grafts. The antiserum (A.K.P.) was prepared against cancer plasma. The two cancer plasmas give a strong band of precipitate which is almost absent from the normal rat plasma (N).

FIG. 3.—1-day Walker tumour rat plasma (K) contrasted with normal rat plasma (N). The cancer plasmas show a strong band in mid-spectrum which in the normal is diffuse. The line ahead of this is also stronger in the cancer blood, and there are other differences.

FIG. 4.—4-day Yoshida sarcomar at plasma (K) contrasted with normal rat plasma (N) to show the stronger mid-line of the cancer plasma.

FIG. 5.— 16-day August tumour plasma (K) showing a much stronger middle band than the normal (N). The antiserum (A.K.P.) was here used in a dilution of 1 : 1.

FIG. 6.—Plasma from rats bearing embryonic liver grafts for 4 days (E) contrasted with a 4-day Walker tumour plasma (K) and a control plasma from a rat with benzpyrene implanted (BP) which was negative. The bottom (E) plasma is almost as strong in the antigen as the Walker plasma.

FIG. 7.—Plasma from rats 8 days after partial hepatectomy (Hz) is contrasted with normal rat plasma (N). The mid-band is much stronger than the normal in one of the hepatectomy plasmas, but only slightly stronger in the other.

FIG. 8.—Plasma from rats pregnant for 10 and 20 days is contrasted with the normal.

FIG. 9.—Normal rat serum (N) and 13-day Walker tumour serum (K) are placed in the top wells to show the position of the antigen line under study. It is much stronger in the K serum. The third well contains a mucoprotein preparation of 13-day Walker tumour serum (M.P.). It gives 3 main lines, at least one of which is seen to arc with the strong antigen of the K serum.

K
N
A.A.K.P
1

K
N
A.K.P.
N
K
2

K
N
A.K.P.
N
K
3

K
N
A.K.P.
N
K
4

Hz
N
A.K.P.
N
Hz
7

K
N
A.K.P.
N
K
5

Preg.
10d.
N
A.K.P.
N
Preg.
20d.
8

BP
E
A.K.P.
E.
K
6

N
K
A.K.P.
M.P.
9

It seems very likely therefore, that the increase in this substance is associated with active tissue growth rather than with a phenomenon such as tissue necrosis. For the necrosis hypothesis does not explain the negative results obtained with the majority of normal tissue grafts which certainly become necrotic. Nor could necrosis account for the positive results with pregnancy, regeneration, and 1-week-old rat plasma. On the other hand the increase of the substance appears to be related to the growth rate of the various tumours and to the extent and duration of the growth of normal tissue. The results suggest that the fundamental correlation might be with the overall mitotic rate of the body. The number of mitoses in a Walker tumour at 24 hours after transplantation could well be as great as in a much larger and later August tumour, or in a regenerating liver lobe. The failure to obtain a significant increase of the substance with most normal tissue grafts could be explained by their relatively low growth rates and the small amount of tissue involved. The level of the substance in normal blood could be accounted for by the mitotic activity needed for normal growth, maintenance, and repair. In short, the hypothesis that the concentration of the substance in the blood is directly associated with the total mitotic activity of the body seems worth considering.

It is more probable, however, that the fundamental correlation is with a secondary effect of tissue growth. Since the substance is apparently a mucoprotein, it may be merely an expression of connective tissue activity. Connective tissue activity cannot be ruled out in any of the experimental situations described. In fact it is difficult to rule it out for any tissue growth *in vivo*. It is worth observing, however, that the high level of the substance in the blood during liver regeneration appears to be out of proportion to the visible fibroblast activity. The actual laparotomy can only account for a minor part of the increase.

The fact that the substance appears to be an $\alpha$-globulin and a mucoprotein raises the question of its identity or otherwise with similar substances reported by other authors. Numerous reports are to be found in the literature of increases of $\alpha$-globulins and mucoproteins in cancer and other conditions. Butler (1951) working in this laboratory on Brdička's (1939) polarographic serum test found that this method of testing human serum, although useless as a diagnostic tool for cancer because of the high proportion of false positions it gave with other diseases, was nevertheless useful for predicting the course of the disease. A serum mucoprotein appeared to be responsible for the polarographic behaviour (Winzler and Smyth, 1948), and this protein appeared to migrate with the $\alpha$-globulins (Boyland, Butler and Conway, 1951). It appears likely therefore that the substance dealt with in the present paper may be the analogous substance in the rat to that dealt with in man by the polarographic method. However, since sulphosalicylic acid deproteinization of rat serum left at least three antigens, only one of which undergoes an increase during tissue growth and is the subject of the present paper, it seems that the gel diffusion method is more highly selective and could give more accurate results.

Shetlar (1952) states that the polysaccharide content of the albumin fraction of serum appears to be elevated in all cases in which tissue proliferation occurs but that the polysaccharide content of the $\alpha$-globulin fraction increases in fever. This is not in accord with the present results.

Hokkanen, Pyörälä and Taipali (1956) studying the serum mucoprotein of rats bearing on ascites tumour found that it reached its maximum after the tumour began to regress. This argues against the cell-division hypothesis proposed earlier, but it may be remarked (*a*) that besides the present antigen there are other mucoprotein components in rat serum which might be responsible for the high level and (*b*) it cannot be ruled out at once that there is less overall cell-division going on in the body after the tumour begins to regress—the activity of normal tissues, especially lymphoid and myeloid tissue, has to be taken into account.

Lockey, Anderson and Maclagan (1956) in a survey of the mucoprotein content of serum and urine report that high levels of these substances are frequently found in inflammation and in the collagen diseases as well as in cancer. Furthermore, surgical operations almost always produced a marked rise. In the light of the present results it is interesting to consider whether this rise in human serum mucoprotein might be due to one mucoprotein only, so that if this substance alone could be followed greater precision would result.

A recent report which is of great interest in the light of present findings is that of Nisselbaum and Bernfeld (1956). These authors isolated the $\alpha$-globulin glycoproteins of the plasma of normal mice and tumour-bearing mice. The substances were studied chemically and physically and found to be distinct from one another. The protein preparation from the tumour-bearing animals behaved on electrophoresis like a nearly uniform substance over a wide range of pH, while that from normal mice was a mixture of several proteins. The authors conclude, " it appears likely that the $\alpha$-globulin isolated from tumour-bearing mice is one of the regular constituents of normal mouse plasma $\alpha$-globulin, the concentration of which increases during tumour growth ". The present findings in the rat are in harmony with this statement. On the other hand the earlier suggestion of Bernfeld and Homberger (1955) based on negative results obtained with normal and embryonic tissue grafts, that the increase of the plasma $\alpha$-globulin is specifically associated with neoplastic growth, is opposed by the present results. It was found that if the growth of normal tissue was sufficiently vigorous, then positive tests were obtained.

Finally, there are one or two questions concerning the gel diffusion results that require discussion. The first is about certain assumptions made in interpretation of the precipitin lines. The assumption was made throughout that since the antigen under study gave a single line it was therefore a single substance. This need not necessarily be so. But since a wide range of antigen dilutions failed to split the line into components, the singleness of the antigen must be assumed until there is good evidence to the contrary.

The other assumption was that if a line is found to be nearer the antiserum well than another line with which it gives a " reaction of identity ", that the antigen responsible is in higher concentration in the first case. This is usually a safe assumption. However, if the first antigen should be of lower molecular weight (e.g. because it was partly hydrolysed) and therefore diffused faster than the other, the position-difference might be obtained without any actual increase of antigen. The author has had exactly this experience with the fibrinogen line of rat plasma which had become infected. However, in this eventuality the faster-diffusing antigen would be expected to give a line of different density and appearance, if not a reaction of " partial identity ". Since there was no evidence for this, the more straightforward interpretation was assumed here.

There were other antigenic changes noted in early tumour blood and it is hoped to present a fuller study of these at a later date.

### SUMMARY

An attempt has been made to discriminate the blood plasma of rats bearing very early tumours from that of control rats. The method used was the analysis of plasma antigens by means of the gel-diffusion technique of Ouchterlony (1948).

No antigenic substance was found in the plasma of rats bearing early tumours which was not also present in the control plasma. However, one striking quantitative difference is reported ; namely an antigenic substance in normal blood which undergoes a large increase in concentration during tumour growth. Twenty-four hours after implantation of the Walker tumour this increase is approximately five-fold. When the slow-growing August tumour was employed no substantial increase was observed until after 8 to 16 days following transplantation. During benzpyrene carcinogenesis the substance had already increased in the blood when the earliest tumours were present.

Further experiments showed that the phenomenon occurred in non-cancerous rats which possessed much actively-growing tissue, e.g. in animals regenerating liver, kidney or skin, and also in pregnant females. The substance was found in distinctly higher concentration in the blood of 1-week-old rats than in that of 8-week-old rats.

The substance appears to be a mucoprotein which migrates electrophoretically with the $\alpha$-globulins. Its relation to similar substances reported on the literature is discussed.

The working hypothesis is suggested that the substance is directly associated with cell-division.

The author is much indebted to Dr. B. Cinader who carried out the immune electrophoresis, to Professor E. Boyland for advice on biochemical procedure and to Miss Teresa Scruton for outstanding technical assistance.

This investigation was supported by grants to the Royal Cancer Hospital and Chester Beatty Research Institute from the British Empire Cancer Campaign, the Jane Coffin Childs Memorial Fund for Medical Research, the Anna Fuller Fund and the National Cancer Institute of the National Institutes of Health, U.S. Public Health Service.

### REFERENCES

BERNFELD, P. AND HOMBURGER, F.—(1955) *Cancer Res.*, **15**, 359.

BOYLAND, E., BUTLER, L. O. AND CONWAY, B. E.—(1951) *Brit. J. Cancer*, **5**, 235.

BRDIČKA, R.—(1939) *Nature*, **139**, 330.

BUTLER, L. O.—(1951) *Brit. J. Cancer*, **5**, 225.

DARCY, D. A.—(1955) *Nature*, **176**, 643.

GRABAR, P. AND WILLIAMS, J. A.—(1953) *Biochim. biophys. Acta*, **10**, 193.

HOKKANEN, E., PYÖRÄLÄ, K. AND TAIPALE, E.—(1956) *Acta path. microbiol. scand.*, **39**, 15.

LOCKEY, E., ANDERSON, N. J. AND MACLAGAN, N. F.—(1956) *Brit. J. Cancer*, **10**, 209.

NISSELBAUM, J. S. AND BERNFELD, P.—(1956) *J. Amer. chem. Soc.*, **78**, 687.

OUCHTERLONY, Ö.—(1948) *Ark. Kemi. Min. Geol.*, **26B**, 1.

SHETLAR, M. R.—(1952) *Texas Rep. Biol. Med.*, **10**, 228.

WINZLER, R J. AND SMYTH, I. M.—(1948) *J. clin. Invest.*, **27**, 617.